# Precision Oncology in Ocular Melanoma: Integrating Molecular and Liquid Biopsy Biomarkers

**DOI:** 10.3390/cimb48020131

**Published:** 2026-01-25

**Authors:** Snježana Kaštelan, Fanka Gilevska, Zora Tomić, Josipa Živko, Tamara Nikuševa-Martić

**Affiliations:** 1 Clinical Hospital Dubrava, Department of Ophthalmology, School of Medicine, University of Zagreb, 10000 Zagreb, Croatia; 2 Promedika Ophthalmology Hospital, 1000 Skopje, North Macedonia; 3 Health Centre of the Croatian Department of Internal Affairs, 10000 Zagreb, Croatia; 4 Clinical Hospital Merkur, Clinical Department of Diagnostic and Interventional Radiology, 10000 Zagreb, Croatia; 5 Department of Biology and Genetics, School of Medicine, University of Zagreb, 10000 Zagreb, Croatia

**Keywords:** ocular melanoma, uveal melanoma, conjunctival melanoma, molecular biomarkers, liquid biopsy, precision oncology, circulating tumour DNA, minimal residual disease (MRD), prognostic biomarkers, translational oncology

## Abstract

Ocular melanomas, comprising uveal melanoma (UM) and conjunctival melanoma (CoM), represent the most common primary intraocular and ocular surface malignancies in adults. Although rare compared with cutaneous melanoma, they exhibit unique molecular landscapes that provide critical opportunities for biomarker-driven precision medicine. In UM, recurrent mutations in GNAQ and GNA11, together with alterations in BAP1, SF3B1, and EIF1AX, have emerged as key prognostic biomarkers that stratify metastatic risk and guide surveillance strategies. Conversely, in CoM, the mutational spectrum overlaps with cutaneous melanoma, with frequent alterations in BRAF, NRAS, NF1, and KIT, offering actionable targets for personalised treatment. Beyond genomics, epigenetic signatures, microRNAs, and protein-based markers provide further insights into tumour progression, microenvironmental remodelling, and immune evasion. In parallel, liquid biopsy has emerged as a minimally invasive approach for real-time disease monitoring. Analyses of circulating tumour DNA (ctDNA), circulating tumour cells (CTCs), and exosome-derived microRNAs demonstrate increasing potential for early detection of minimal residual disease, prognostic assessment, and evaluation of treatment response. However, the clinical integration of these biomarkers remains limited by tumour heterogeneity, technical variability, and the lack of unified translational frameworks. This review synthesises current knowledge of molecular and liquid biopsy biomarkers in ocular melanoma, highlighting their relevance for diagnosis, prognosis, and treatment personalisation. The integration of established tissue-based molecular markers with novel liquid biopsy technologies will enable a unique framework for biomarker-guided precision oncology and risk-adapted surveillance in uveal and conjunctival melanoma, offering insight into strategies for early detection, therapeutic monitoring, and personalised clinical management.

## 1. Introduction

Ocular melanomas, comprising uveal melanoma (UM) and the less common conjunctival melanoma (CoM), represent the most frequent primary intraocular and ocular surface malignancies in adults [1,2,3,4]. Although often grouped clinically, UM and CoM are biologically distinct entities with divergent epidemiology, molecular architecture, and clinical behaviour [2,4,5,6]. UM accounts for 85–90% of ocular melanomas and most commonly arises in the choroid, followed by the ciliary body and iris [7,8,9]. Despite high rates of local tumour control, approximately 50% of patients ultimately develop metastatic disease, predominantly hepatic, with a median survival of less than one year [10,11,12]. Although significant advances have been made in genomic profiling, current surveillance strategies, including ultrasound, optical coherence tomography (OCT), magnetic resonance imaging (MRI), and tissue biopsy, remain limited in their ability to detect early metastatic dissemination or dynamic tumour evolution [1,8,13,14,15].

A major unmet clinical need is the early detection of minimal residual disease (MRD), which refers to microscopic, radiologically occult dissemination that precedes clinically evident metastasis [16,17]. The inability of conventional imaging to detect MRD contributes to delays in diagnosis and drives the development of sensitive molecular and liquid biopsy approaches capable of identifying subclinical tumour activity [18,19].

CoM, although far less common, exhibits aggressive behaviour with substantial risks of local recurrence and systemic spread [3,5,20]. In contrast to UM, CoM is strongly associated with ultraviolet radiation and shares key genetic features with cutaneous melanoma [2,21].

UM is characterised by a particular mutational landscape. Activating mutations in G protein subunit alpha q (*GNAQ*) and G protein subunit alpha 11 (*GNA11*), present in ~90% of cases, initiate oncogenesis by constitutively activating the protein kinase C (PKC) and mitogen-activated protein kinase/extracellular signal-regulated kinase (MAPK/ERK) pathways [6,22,23]. However, metastatic progression is primarily determined by secondary alterations. Loss-of-function mutations in BRCA1-associated protein 1 (*BAP1*) are strongly associated with early metastasis and the high-risk Class 2 gene expression profile. Mutations in splicing factor 3b subunit 1 (SF3B1) predict late-onset metastasis, and mutations in eukaryotic translation initiation factor 1A, X-linked (EIF1AX) correlate with favourable outcomes [5,6,24,25,26,27].

In contrast, CoM closely mirrors cutaneous melanoma, with frequent mutations in B-Raf proto-oncogene (*BRAF*), neuroblastoma RAS viral oncogene homolog (*NRAS*), neurofibromin 1 (*NF1*), and KIT proto-oncogene (*KIT*), enabling the application of targeted and immune-based therapies [3,6,26,28,29].

Beyond genomic events, epigenetic signatures, DNA methylation patterns, microRNAs (miRNAs), long non-coding RNAs (lncRNAs), and proteomic biomarkers provide additional insight into tumour progression, microenvironmental modulation, and immune evasion [25,30,31,32,33,34,35]. However, conventional tissue-based profiling offers only a static snapshot of tumour biology and is limited by tumour heterogeneity and the risks associated with ocular biopsy.

These limitations have fuelled growing interest in liquid biopsy technologies, including circulating tumour DNA (ctDNA), circulating tumour cells (CTCs), and exosome-derived miRNAs, which enable minimally invasive, real-time tumour monitoring [15,18,19,25,36,37,38]. In UM, ctDNA has demonstrated potential for early MRD detection, clonal evolution assessment, and prediction of therapeutic response [16,17]. Although liquid biopsy in CoM is less studied, early evidence suggests a role in early metastasis detection, molecular profiling, and treatment stratification [3,19,39].

Despite these advances, translating molecular and circulating biomarkers into routine management remains limited by technical heterogeneity, challenges with assay standardisation, and the rarity of ocular melanoma, which complicates large-scale validation [3,17,25,33,39,40,41,42,43].

Taken together, the field is shifting toward biomarker-driven precision oncology, in which molecular profiling and liquid biopsy may enable personalised diagnostic, prognostic, and therapeutic strategies for both UM and CoM [3,16,18,19,39,44]. In this context, this review brings together current evidence on tumour-intrinsic molecular alterations and circulating biomarkers, emphasising their complementary roles in diagnosis, prognostic stratification, and therapeutic decision-making. By integrating tissue-based profiling with emerging liquid biopsy approaches, it highlights how such biomarker convergence may support more personalised, risk-adapted surveillance strategies, with particular relevance for MRD detection in ocular melanoma.

### Data Collection

A comprehensive narrative literature review was conducted to provide an integrative and up-to-date overview of molecular and liquid biopsy biomarkers in ocular melanoma, with a particular focus on UM and CoM. The primary objectives were to summarise current evidence on genetic, epigenetic, transcriptomic, proteomic, and circulating biomarkers, and to highlight their diagnostic, prognostic, predictive, and translational relevance in precision oncology.

A systematic literature search was conducted using the PubMed/MEDLINE and Google Scholar databases, encompassing publications available up to December 2025. The search strategy employed combinations of the following keywords: “ocular melanoma”, “uveal melanoma”, “conjunctival melanoma”, “molecular biomarkers”, “genetic biomarkers”, “genomic profiling”, “GNAQ”, “GNA11”, “BAP1”, “SF3B1”, “EIF1AX”, “chromosomal aberrations”, “epigenetic biomarkers”, “DNA methylation”, “RNA methylation”, “microRNA”, “non-coding RNA”, “long non-coding RNA”, “proteomic biomarkers”, “tumour microenvironment”, “liquid biopsy”, “circulating tumour DNA”, “ctDNA”, “circulating tumour cells”, “CTCs”, “extracellular vesicles”, “exosomes”, “minimal residual disease”, “precision oncology”, and “personalised medicine”.

The search was limited to articles published in the English language. Following initial identification, duplicate records were removed, and titles and abstracts were screened for relevance. Full-text articles meeting the inclusion criteria were subsequently reviewed in detail. Reference lists of selected publications were manually screened using a snowballing approach to identify additional relevant studies.

All types of peer-reviewed scientific publications were considered, including original preclinical and clinical studies, systematic reviews, and meta-analyses. Both human and relevant experimental studies were included where appropriate. Emphasis was placed on high-quality studies published within the past five years to ensure inclusion of the most current evidence, while earlier publications were retained when essential for contextual completeness. Articles outside the scope of this review, those without English translations, and studies of limited methodological quality were excluded. No quantitative data synthesis or meta-analysis was performed, as this review was designed as a narrative synthesis integrating mechanistic and clinical evidence.

## 2. Molecular Biomarkers in Uveal Melanoma

UM is a rare but highly aggressive intraocular malignancy originating from melanocytes within the uveal tract. It primarily arises in the choroid (~90%), followed by the ciliary body (7%) and iris (3%). Despite advances in local tumour management, approximately 50% of patients develop metastatic disease, predominantly involving the liver, with a median survival of often less than one year [10,11,12].

### 2.1. Mutational Landscape: GNAQ, GNA11, BAP1, SF3B1, and EIF1AX

The molecular pathogenesis of UM is characterised by a distinct mutational profile from that of cutaneous melanoma. Activating mutations in GNAQ or GNA11 occur in approximately 80–85% of UM tumours and constitute the initiating oncogenic event. These mutations lock Gα subunits in a GTP-bound state, leading to continuous activation of the PKC, MAPK/ERK, and YAP/TAZ signalling pathways, which promote tumour growth and survival [22,23]. Consequently, *GNAQ* and *GNA11* are considered early driver mutations, essential for tumour initiation but not sufficient on their own to induce metastasis.

Piaggio et al. reported that patients harbouring *GNA11* mutations experienced shorter disease-specific survival than those with *GNAQ* mutations, and tumours with mixed *GNAQ*/*GNA11* mutational profiles tend to have worse outcomes [45,46].

Progression to metastatic disease is primarily fuelled by secondary genetic alterations, with BAP1 mutations emerging as the most frequent and influential driver. Located on chromosome 3, *BAP1* encodes a deubiquitinating enzyme that plays pivotal roles in cell cycle regulation, differentiation, DNA repair, and transcriptional control [47]. Loss of *BAP1* function, through mutation or deletion, is the most potent molecular predictor of metastasis [27]. Germline mutations in *BAP1* underlie *BAP1* tumour predisposition syndrome (BAP1-TPDS), which confers increased risk for UM, cutaneous melanoma, mesothelioma, and renal cell carcinoma [48]. Harbour et al. demonstrated that monosomy 3, combined with inactivation of the remaining *BAP1* allele, confers a markedly higher metastatic potential [22].

Two additional secondary mutations, *EIF1AX* and splicing factor 3B subunit 1 (*SF3B1*), further refine prognostic assessment in UM. The *EIF1AX* gene encodes a protein involved in translation initiation by stabilising ribosomal recognition of the start codon [49]. Tumours with *EIF1AX* mutations tend to have a low metastatic risk and are associated with favourable survival outcomes [50]. Conversely, mutations in *SF3B1* are associated with an intermediate-risk phenotype and are characteristically linked to late-onset metastatic disease, often occurring more than five years after initial diagnosis [49,51]. Together, these mutations define the molecular and genetic landscape of UM [52,53,54].

Table 1 presents a structured overview of the key molecular biomarkers in UM, highlighting their functional roles, prognostic significance, and translational relevance [1,6,10,14,22,23,24,25,40,45,46,47,48,49,50,51,52,53,54,55,56]. UM is characterised by a limited set of early driver mutations, accompanied by secondary alterations that collectively play a critical role in determining metastatic risk.

### 2.2. Chromosomal Aberrations (Monosomy 3, 8q Gain)

Chromosomal aberrations are among the strongest prognostic biomarkers in UM. The two most clinically relevant alterations, monosomy three and 8q gain, are closely associated with metastatic progression. Monosomy 3 is strongly associated with BAP1 loss and defines the highest-risk subgroup of UM, with markedly increased likelihood of early systemic dissemination [22,48]. In contrast, 8q gain is frequently observed alongside monosomy three and further amplifies metastatic potential by promoting tumour growth, chromosomal instability, and transcriptional reprogramming [24,55]. Tumours displaying both monosomy 3 and 8q gain constitute the most aggressive molecular subtype, whereas disomy 3 and absence of 8q gain correlate with favourable outcomes. These cytogenetic biomarkers, therefore, serve as essential components of modern risk-stratification frameworks in UM [56].

### 2.3. Prognostic Significance and Patient Stratification

Cytogenetic abnormalities further enhance prognostic precision in UM. Monosomy 3, gain of 8q, and losses at 1p or 8p are strongly associated with increased metastatic potential [55]. By integrating these chromosomal abnormalities with specific gene mutations, UM can be classified into clinically meaningful molecular subtypes, providing more precise risk stratification [24,48,56].

One of the most widely used clinical assays is Genome-Wide Expression Profiling (GEP), which categorises UMs into three prognostic classes. Class 1 tumours (low-risk), which are further divided into Class 1A and Class 1B, with corresponding 5-year metastatic risks of 2% and 21%, and Class 2 tumours (high-risk), which carry a 5-year metastatic risk of approximately 72%. Importantly, the prognostic power of the Class 2 GEP signature exceeds that of many conventional clinicopathologic and genetic markers, whether considered individually or in combination [48,56]. 

Recent research has defined four molecular-clinical subgroups (1–4 or A–D) that combine gene mutations with chromosomal changes. Class 1/A (disomy 3, *EIF1AX* mutation, 6p gain) shows a favourable prognosis. Class 2/B (disomy 3 with *SF3B1* or *SRSF2* mutations and 6p or 8q gain) carries an intermediate risk of late metastasis. Class 3/C (monosomy 3 plus *SF3B1*, *SRSF2*, or *BAP1* mutations and 8q gain) is linked to poor outcomes. Class 4/D (monosomy 3, *BAP1* mutation, 8q gain, and/or multiple copy-number alterations) has the highest metastatic potential and worst survival [24].

These integrated classification models refine prognostic assessment by contextualising established mutation-specific risk profiles within broader transcriptomic and cytogenetic frameworks. Consequently, molecular profiling has become an essential component of prognostic evaluation and treatment planning in UM [6,24].

### 2.4. Implications for Targeted and Immunotherapy-Based Treatment

Molecular characterisation not only refines prognosis but also guides the development of precision therapies. Although central to tumour initiation, GNAQ and GNA11 mutations currently lack clinically effective direct inhibitors [23]. Nonetheless, combination approaches targeting multiple signalling nodes are still being explored.

In contrast, *BAP1* loss provides both prognostic and therapeutic insights. Beyond its association with metastasis, *BAP1* deficiency contributes to immune evasion, epigenetic dysregulation, and alteration in the tumour microenvironment. These findings suggest that *BAP1*-deficient tumours may respond favourably to immune-modulating therapies, including T-cell receptor-directed agents such as tebentafusp, which has demonstrated improved survival in human leukocyte antigen (HLA) A2.01-positive patients [57,58,59].

Clinical trials investigating targeted therapies for UM have explored several molecular approaches, including inhibitors of mitogen-activated protein kinase (MEK), phosphoinositide 3-kinase/AKT serine/threonine kinase/mechanistic target of rapamycin (PI3K/AKT/mTOR), histone deacetylases (HDAC), and inhibitors of the bromodomain and extraterminal protein family, as well as tyrosine kinase receptor MET and multi-tyrosine kinase inhibitors. These treatments aim to interfere with signalling pathways activated by driver mutations, as well as with epigenetic and transcriptional mechanisms involved in tumour progression [60]. However, the biological divergence between cutaneous melanoma and UM necessitates distinct therapeutic strategies, as treatments effective in cutaneous melanoma often fail in UM due to fundamental differences in their genomic landscapes and responses to targeted agents [61,62].

The integration of genetic, cytogenetic, and transcriptomic data is transforming UM management from a uniform treatment paradigm to a precision oncology approach. While early driver mutations (*GNAQ/11*) initiate tumour development, secondary mutations (*BAP1, SF3B1, EIF1AX*) and chromosomal aberrations determine metastatic potential and therapeutic responsiveness. Understanding tumour heterogeneity and clonal evolution further refines patient selection for intensified surveillance or enrolment in a clinical trial [6,63,64].

Ultimately, comprehensive molecular assessment now forms the cornerstone of modern UM care, linking genotype to clinical phenotype and enabling individualised strategies for surveillance, systemic therapy, and immunotherapeutic intervention.

## 3. Molecular Biomarkers in Conjunctival Melanoma

CoM is an uncommon but clinically significant ocular surface malignancy arising from melanocytes of the conjunctival epithelium. Despite its rarity, CoM demonstrates aggressive biological behaviour with a substantial risk of local recurrence and metastatic spread, particularly to regional lymph nodes, lungs, and liver. Importantly, unlike UM, CoM shares considerable molecular resemblance to cutaneous melanoma, which has profoundly influenced its current therapeutic landscape. Since cutaneous melanoma has been extensively studied and treated using targeted and immune-based therapies, advances in its biology have enabled the translation of biomarker-driven precision oncology into CoM management [2,3,21,28]. This genetic similarity to cutaneous melanoma, particularly in the prevalence of mutations in the *BRAF* and *NRAS* genes, significantly distinguishes CoM from UM, which rarely harbours these mutations [62].

A central molecular hallmark of CoM is the high prevalence of mutations that activate the MAPK pathway. This signalling cascade, comprising the RAS/RAF/MEK/ERK modules, regulates key cellular processes including proliferation, differentiation, and survival. Oncogenic activation of MAPK signalling promotes uncontrolled cellular growth and malignant transformation [65,66]. In addition, dysregulation of the PI3K/AKT/mTOR pathway contributes to tumour progression, metastatic potential, and therapy resistance. Crosstalk between MAPK and PI3K signalling underscores the complexity of CoM biology and highlights the importance of identifying pathway-specific biomarkers.

Table 2 summarises the key molecular biomarkers in CoM, highlighting alterations in the MAPK and PI3K/AKT pathways and their potential therapeutic relevance [2,3,21,28,65,66,67,68,69,70,71,72,73,74,75,76,77,78,79].

### 3.1. BRAF Mutations

BRAF mutations are the most frequently identified genetic alteration in CoM, reported in approximately 30–50% of cases across published cohorts [68,69]. Mutations in the BRAF gene, most commonly the V600E substitution, lead to constitutive activation of the MAPK pathway, driving oncogenesis [66,70], especially in tumours arising on sun-exposed areas of the conjunctiva [65,70]. While these mutations are strongly oncogenic, they are not consistently associated with adverse clinical outcomes and currently serve primarily as predictive biomarkers for response to MAPK-targeted therapy [66,70]. Therefore, BRAF status is not yet considered an established prognostic biomarker.

However, the therapeutic relevance of BRAF mutations is compelling. BRAF inhibitors, alone or in combination with MEK inhibitors, are standard treatment options in cutaneous melanoma and have shown promising efficacy in selected cases of CoM. Brouwer et al. reported tumour regression in patients receiving BRAF and/or MEK inhibitors, providing early evidence of clinical benefit [28]. Combination therapy is preferred due to the development of resistance to BRAF monotherapy, a pattern also observed in cutaneous melanoma. As experience grows, BRAF mutational status may evolve into a combined prognostic and predictive biomarker for selection of targeted therapy [3].

### 3.2. NRAS Mutations

*NRAS* encodes a guanosine triphosphate (GTP) binding protein that regulates MAPK and PI3K/AKT signalling. *NRAS* mutations occur in approximately 20% of CoMs and are generally mutually exclusive with *BRAF* mutations, reflecting distinct molecular subtypes. In contrast to *BRAF*-mutant tumours, *NRAS*-mutant tumours may be associated with less favourable outcomes in some cohorts [21,65]. Given their role in MAPK signalling, *NRAS*-mutant tumours may be amenable to MEK inhibitor therapy, although clinical evidence in CoM remains limited. The mutually exclusive occurrence of *BRAF* and *NRAS* mutations, present in many but not all CoMs, underscores the tumour’s molecular heterogeneity and the need for personalised molecular profiling to inform therapeutic decisions [3,71].

### 3.3. NF1 Mutations

The *NF1* gene encodes neurofibromin, a tumour suppressor protein that negatively regulates *RAS* activity, thereby acting as a brake on the MAPK pathway. Loss-of-function mutations in NF1 result in sustained MAPK pathway activation and are reported in approximately 30–40% of CoM. Approximately one-third of CoM cases carry *NF1* mutations [21,65,72]. Unlike *BRAF* and *NRAS* mutations, *NF1* mutations may coexist with other driver alterations, including *BRAF* and *NRAS* [73,74]. NF1-mutant tumours often exhibit a higher tumour mutational burden, which may boost immunogenicity and improve responses to immune checkpoint inhibition [72,75,76]. Thus, *NF1* mutation status may serve as a biomarker for predicting immunotherapy response rather than direct targeted therapy.

### 3.4. KIT Alterations

*C-KIT* encodes a receptor tyrosine kinase involved in MAPK signalling and other downstream signalling pathways. *C-KIT* mutations or amplifications are typically mutually exclusive of *BRAF* and *NRAS* mutations, although rare cases of coexistence have been described [3,65,66,72,74]. Tyrosine kinase inhibitors such as imatinib or dasatinib have demonstrated benefit in *C-KIT* mutant cutaneous melanoma, and case reports suggest potential applicability in selected CoM patients. Although rare, KIT alterations are clinically relevant since they create opportunities for targeted therapy in selected patients [3,74,77].

### 3.5. TERT Promoter Mutations

Recent genomic profiling has identified recurrent mutations in the telomerase reverse transcriptase (TERT) promoter region in CoM, particularly the C228T and C250T variants [77]. These mutations upregulate telomerase activity, promoting cellular immortality and tumour progression. Their presence appears more frequent in tumours associated with chronic ultraviolet exposure, providing a mechanistic link between UV damage and carcinogenesis. TERT promoter mutations have been proposed as markers of more aggressive tumour behaviour. The incorporation of TERT mutation testing into molecular workups may improve risk stratification, particularly when combined with *BRAF, NRAS*, or *NF1* status [77,78].

### 3.6. Immune Checkpoint Pathways and Immunological Biomarkers

Beyond oncogenic drivers, CoM exhibits immune-evasive mechanisms through checkpoint pathways, including cytotoxic T-lymphocyte antigen 4 (CTLA-4) and programmed cell death protein 1 (PD-1). Immune checkpoint inhibitors have revolutionised the management of advanced cutaneous melanoma and are increasingly adopted in CoM [3,79]. Gkiala et al. reported complete tumour regression in 8 of 13 CoM patients treated with checkpoint blockade, demonstrating substantial therapeutic potential [67].

Multiple immune-related biomarkers may guide treatment selection. PD-L1 expression in tumour cells and tumour-infiltrating lymphocytes has been associated with response to immunotherapy in cutaneous melanoma and is now being explored in CoM. Additionally, tumours with high tumour mutational burden (TMB) and high neoantigen load tend to be more immunogenic and may respond more favourably to checkpoint blockade. *NF1*-mutant CoM, which often exhibits an elevated mutational burden, may therefore represent an immunologically responsive subtype [72,76].

Although microsatellite instability (MSI) is rare in CoM, MSI-high tumours generally display enhanced immunotherapy sensitivity across cancer types, supporting the rationale for MSI testing in atypical or treatment-refractory cases. The integration of these emerging immune biomarkers with liquid biopsy approaches, such as ctDNA and exosomal miRNAs, could enable real-time, dynamic monitoring of immunotherapy responses in the future [80,81].

### 3.7. Comparative Genomics of Conjunctival and Uveal Melanoma

While MAPK pathway activation is frequent in CoM, it is rare in UM. Populo et al. reported MAPK activation in 86% of cases of CoM [82]. In contrast, UM is driven primarily by mutations in *GNAQ* and *GNA11*, as well as secondary alterations in *BAP1*, *SF3B1*, and *EIF1AX* [83]. These molecular differences explain divergent therapeutic outcomes: BRAF/MEK inhibitors and checkpoint blockade have shown efficacy in cutaneous melanoma and CoM, but not in UM [84]. This contrast highlights the significance of genetic profiling in distinguishing ocular melanoma subtypes, predicting therapeutic responses, and selecting biomarker-guided interventions.

The molecular characterisation of CoM has revealed a complex landscape dominated by alterations in MAPK and PI3K/AKT/mTOR signalling pathways. *BRAF*, *NRAS*, *NF1*, and *C-KIT* mutations not only illuminate disease pathogenesis but also provide opportunities for targeted and immune-based therapies. Although prognostic associations remain limited for some biomarkers, their therapeutic relevance underscores the central role of molecular profiling in CoM management. However, tumour behaviour is also shaped by epigenetic regulation, transcriptomic signatures, and protein expression profiles. These additional biomarker classes offer further insights into tumour progression, microenvironmental modulation, and therapeutic vulnerability. Notably, the overlap between CoM and cutaneous melanoma allows translation of established therapies, including BRAF/MEK inhibitors and immune checkpoint blockade, into ocular oncology. Distinct molecular differences from UM, however, emphasise the need for precise genetic characterisation and patient-specific treatment strategies. Moving forward, expanding genomic, transcriptomic, and immunologic profiling in larger, multicentre cohorts will be essential to validate candidate biomarkers, refine prognostic models, and integrate precision oncology into the standard management of CoM [3,6,14,21,85].

## 4. Epigenetic, Transcriptomic, and Proteomic Landscapes in Ocular Melanoma

Epigenetic, transcriptomic, and protein biomarkers extend the genomic landscape of CoM and UM, adding a crucial regulatory layer that influences tumour initiation, progression, metastasis, and therapeutic response. Aberrant DNA methylation, histone modifications, RNA methylation, dysregulated non-coding RNAs, and proteomic alterations have emerged as reliable biomarkers for diagnosis, prognosis, and disease monitoring, while simultaneously revealing actionable therapeutic vulnerabilities. These markers can stratify metastatic risk, discriminate benign from malignant lesions, and pinpoint pathways amenable to targeted or immunomodulatory therapy, thereby supporting personalised oncology for both CoM and UM patients [3,6,30,86].

### 4.1. DNA Methylation, RNA Methylation, and Histone Modifications

Beyond genetic alterations, epigenetic mechanisms play a central role in regulating gene expression, tumour plasticity, and metastatic potential in ocular melanoma. DNA and RNA methylation patterns, together with histone modifications, provide additional layers of prognostic and therapeutic information that complement genomic profiling [3,6,25,30,31,32,33,34,35,86,87,88,89].

#### 4.1.1. Conjunctival Melanoma

Although limited data are available, current evidence suggests that CoM shares global methylation patterns with cutaneous melanoma but differs in promoter-specific regions. Jurmeister et al. reported promoter methylation of several tumour suppressor genes, including APC, CDKN2A, WIF1, and RASSF1, in selected cohorts of CoM. Loss of 5-hydroxymethylcytosine has emerged as a potentially useful epigenetic feature distinguishing melanoma from nevus tissue [87].

RNA methylation further refines tumour phenotypes. Jia et al. reported that reduced methyltransferase-like 3 (METTL3) (“writer”) expression and increased AlkB homolog 5, RNA demethylase (*ALKBH5*) (“eraser”) led to lower m^6^A methylation, which in turn decreased m^6^A-modified histidine triad nucleotide-binding protein 2 (*HINT2*) and correlated with a poor prognosis [88]. He et al. reported that upregulation of beta-site APP-cleaving enzyme 2 (BACE2) mRNA is associated with increased m6A methylation, which promotes melanoma progression via calcium signalling [89]. Although most evidence comes from experimental models, alterations in m^6^A RNA methylation are consistently associated with aggressive biological behaviour [90]. Together, these findings position RNA methylation as a promising therapeutic target for inhibiting tumour growth and angiogenesis.

Histone alterations in CoM are less explored. Enhancer of zeste homolog 2 (EZH2), a histone methyltransferase responsible for gene silencing, is highly expressed in primary CoM and lymph node metastases but absent in normal melanocytes and primary acquired melanosis [74,91,92], suggesting both biomarker and therapeutic potential.

#### 4.1.2. Uveal Melanoma

Epigenetic profiling has been deeply characterised in UM. Bakhoum et al. reported BAP1 promoter methylation as a marker associated with increased metastatic risk in UM [93]. Smith et al. detected aberrant methylation of *BAP1* and *SF3B1*, demonstrating that epigenetic changes arise early and persist in corresponding metastases [94]. Hypermethylation predominantly targets tumour suppressor genes, including cyclin-dependent kinase inhibitor 2A, isoform INK4a (*p16INK4a*), *RASSF1A* and cyclin-dependent kinase inhibitor 2B, isoform INK4b (*p16INK4b*), while hypomethylation is rare [35]. Delta-like ligand 3 (*DLL3*) expression correlates with cytosine–phosphate–guanine (CpG) methylation and is significantly higher in non-metastatic tumours, indicating prognostic relevance [95].

Epigenetic profiling predominantly reveals hypermethylation of tumour suppressor genes, with hypomethylation being rare [96]. The MethylSig-UM classifier enables prognostic stratification of UM based on DNA methylation patterns [97].

### 4.2. Non-Coding RNAs (miRNAs, lncRNAs, circRNAs)

Non-coding RNAs are increasingly recognised as promising biomarkers for diagnosis, prognosis, and disease monitoring. Their inherent stability, accessibility, and tissue-specific expression make them particularly valuable for clinical applications [3,98].

#### 4.2.1. Conjunctival Melanoma

Larsen et al. first reported that reduced expression of miR-204 and miR-211 correlates with an aggressive phenotype and increased risk of progression [70]. Mikkelsen et al. identified miR-21 and miR-146b as markers associated with metastatic dissemination [99]. Ipenberg et al. differentiated cutaneous melanoma from nevi using miRNA signatures, including miR-9-5p, miR-196b-5p, and miR-615-3p, which support their diagnostic utility when biopsy material is limited [78]. Circulating miRNAs regulate a substantial proportion of protein-coding genes and play key roles in tumour biology [98].

#### 4.2.2. Uveal Melanoma

Numerous circulating miRNAs regulate ~60% of protein-coding genes, functioning as tumour suppressors (e.g., miR-9, miR-34a/b/c, miR-124a, miR-145) or oncogenic mediators (miR-20a, miR-21, miR-155, miR-222) [2,6]. Worley et al. linked the upregulation of let-7b, miR-199a-3p/5p, miR-143, and miR-652 to a higher metastatic risk [100], whereas other studies linked miR-149* and miR-134 to liver metastasis [101]. Xin et al. validated multi-miRNA prognostic signatures that stratify UM patients into high- and low-risk groups [102]. Differentially expressed miRNAs also distinguish UM from nevi [103] and from healthy individuals [104]. Collectively, miRNA-based panels hold strong potential for prognostic testing and therapy monitoring in UM [6].

Among long non-coding RNAs (lncRNAs), plasmacytoma variant translocation 1 (*PVT1*) has been identified as upregulated in UM, promoting tumour progression by inhibiting apoptosis and enhancing cell proliferation and migration. Silencing *PVT1* reduces tumour growth, suggesting therapeutic potential [105,106].

### 4.3. Protein Biomarkers: Angiogenesis, Extracellular Matrix, and Immune Regulation

Proteomic changes in the tumour microenvironment complement genomic and epigenetic alterations, influencing angiogenesis, immune escape, and metastatic behaviour.

#### 4.3.1. Angiogenesis and Immune Signalling

The tumour microenvironment governs angiogenesis via a balance of pro- and anti-angiogenic factors. In UM, tumour-associated macrophages polarise towards M2 phenotypes and secrete vascular endothelial growth factor A (VEGF-A), transforming growth factor β (TGF-β), and C-C motif chemokine ligand 2 (CCL2), promoting angiogenesis and metastatic spread [107]. Although CoM is generally hypovascular, VEGF is frequently expressed, and tumour-derived lactate can favour M2 polarisation, thereby increasing the secretion of VEGF, TGF-β, and interleukin-10 (IL-10) [108,109].

Early 8q gain in UM correlates with increased infiltration of M2-polarised macrophages, which drive angiogenesis and lymphangiogenesis and facilitate local, regional, and distant metastasis [110]. Neutrophils further enhance angiogenesis and tumour cell migration [28,111]. Loss of *BAP1* expression likewise augments inflammation, angiogenesis, and T-cell recruitment in UM [112].

#### 4.3.2. Extracellular Matrix Remodelling

UM demonstrates extracellular matrix (ECM) changes, including collagen and fibronectin degradation mediated by matrix metallopeptidase 2 (MMP-2), MMP-14, and tissue inhibitor of metalloproteinases 2 (TIMP-2) [112]. Aughton et al. identified ECM signatures associated with high metastatic risk, including lysyl oxidase-like 3 (LOXL3), LOXL4, collagen type VI alpha chain (Col6A1/2/3), and the hypoxia-regulated prolyl 4-hydroxylase subunit alpha 1 (P4HA1). At the same time, Col4A2 correlates with tumour adhesion and growth [113]. Tenascin-C, an inflammation-associated matrix protein detectable in blood, is upregulated in primary UM and micrometastases and may serve as a minimally invasive prognostic and treatment-monitoring biomarker [114].

#### 4.3.3. Immune Response and Inflammatory Markers

Tumour-associated macrophages, neutrophils, and ECM-related proteins represent candidate biomarkers for disease stratification and novel therapeutic targets [28,57,112,115]. Moreover, the precise mechanisms by which *BAP1* loss contributes to the aggressive metastatic phenotype in UM, particularly its influence on components of the tumour microenvironment such as macrophages and T lymphocytes, remain to be elucidated [62].

Table 3 provides an overview of representative epigenetic, transcriptomic, and protein biomarkers associated with ocular melanoma, summarising their biological functions and potential clinical applications [30,31,34,35,94,95,97,98,99,100,101,104,116]. These biomarkers highlight the regulatory complexity of ocular melanoma, highlighting emerging opportunities for biomarker-guided surveillance and therapeutic strategies. Exosomal and circulating miRNA profiling offer additional diagnostic and prognostic information and may outperform single-analyte approaches when used as biomarker panels. Combining multiple circulating biomarkers, such as ctDNA, miRNAs, and protein signatures, may substantially improve detection accuracy, risk stratification, and treatment monitoring. Integration of these platforms with genomic and immunologic biomarkers is expected to enhance personalised surveillance strategies in ocular melanoma [3,17,33,41,117,118,119].

Collectively, epigenetic and non-coding RNA biomarkers represent a crucial link between static genomic alterations and dynamic liquid biopsy readouts, reinforcing their value for integrated MRD detection and treatment monitoring.

## 5. Liquid Biopsy Biomarkers in Ocular Melanoma

While tissue-based molecular profiling has substantially improved prognostic stratification in ocular melanoma, it remains limited by tumour heterogeneity and its static nature. Liquid biopsy has emerged as a transformative, minimally invasive approach that enables dynamic assessment of tumour burden, clonal evolution, and minimal residual disease [16,17,19,40,42].

### 5.1. Rationale and Advantages of Liquid Biopsy

Liquid biopsy offers a minimally invasive approach to monitor tumour-derived biomarkers longitudinally, with circulating material obtainable from peripheral blood as well as from aqueous or vitreous humour samples [17,40,86,120]. Although routine tumour sampling is standard in CoM, in UM, tumour biopsy carries a risk of bleeding and dissemination, making liquid biopsy particularly valuable [117,121]. For follow-up and metastatic screening, liquid biopsy represents a less invasive and potentially more cost-effective alternative to regular serial imaging [3,86,122]. In contrast to lung, breast, prostate, and colorectal cancer, where multiple Food and Drug Administration (FDA)-approved assays for ctDNA exist, none has yet been approved for UM or CoM [17,86,117,122].

**Figure 1:**
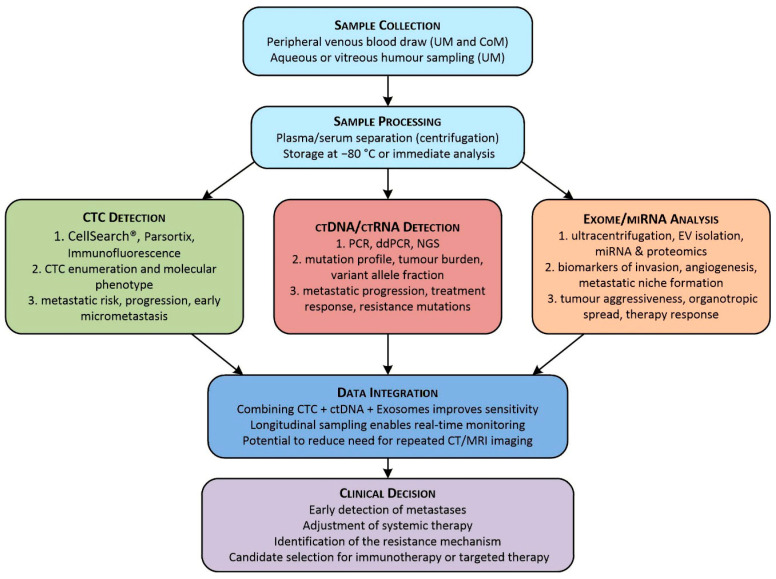
Overview of liquid biopsy platforms in ocular melanoma. A schematic representation of circulating tumour cells (CTCs), circulating tumour DNA (ctDNA), and exosome-derived biomarkers, including their sources, detection methods, and potential clinical applications. 1: 1. methods; 2: output; 3: clinical interpretation; UM: uveal melanoma; CoM: conjunctival melanoma; CTC: circulating tumour cells; ctDNA: circulating tumour DNA; ctRNA: circulating tumour RNA; PCR: polymerase chain reaction; ddPCR: droplet digital PCR; NGS: next-generation sequencing; miRNA: microRNA; EV: extracellular vesicles; CT: computed tomography; MRI: magnetic resonance imaging.

Figure 1 illustrates the workflow of liquid biopsy analysis in ocular melanoma, encompassing CTC-, ctDNA-, and exosome-based approaches [16,17,18,19,33,40,41,118,121,122]. Unlike conventional single-time-point tissue biopsies, liquid biopsy provides a minimally invasive, dynamically evolving method for real-time, longitudinal profiling of ocular melanoma, facilitating continuous monitoring of tumour burden, metastatic progression, and therapeutic response [2,16,42,116,123].

### 5.2. Circulating Tumour Cells

CTCs have established prognostic utility in UM, particularly in metastatic patients, and their quantitative analysis may predict disease progression and overall survival in patients with metastatic disease [17,62]. Advances in single-cell sequencing and sophisticated enrichment techniques are enhancing CTC detection rates and enabling in-depth genomic and transcriptomic analysis, thereby improving their clinical utility for guiding treatment decisions [40].

Detection of CTCs in UM relies on specialised platforms: the FDA-approved CellSearch^®^ system (immunomagnetic capture) and the FDA-approved Parsortix™ system (size- and deformability-based separation), both widely used in UM studies, alongside two investigational size-based and immunofluorescence methods [124,125,126].

The main limitation is antigenic heterogeneity, which complicates the universal identification of CTCs in UM [12]. Marker panels improve sensitivity: Beasley validated gp100+, MART1+, S100β+, CD45-, and CD16– for CTC detection in 86% of primary UM, with shorter overall survival [43,117,127]. Anand et al. introduced the Circulating Melanoma Cell Test (CD146+, HMW-MAA+, CD45−, CD34−, nucleated), detecting CTCs in 60% of cases before radiographic metastasis [128]. However, when CellSearch^®^ was used with a single marker (melanoma-associated chondroitin sulfate proteoglycan), CTC sensitivity was only 30%, while ctDNA was detected in 84% and was strongly associated with progression-free survival [129]. Dual-marker approaches (CD63 + gp100) increased sensitivity up to 91% [130].

Despite promising results, studies remain small and heterogeneous, and there is still no unified multitarget panel for UM since 2008.

### 5.3. Circulating Tumour DNA and RNA

ctDNA analysis has shown promise for identifying MRD and monitoring disease dynamics, enabling early identification of subclinical relapse and dynamic monitoring of treatment response, particularly in high-risk or metastatic UM. Their half-life is measured in hours, enabling near real-time tumour assessment. Detection is performed using polymerase chain reaction (PCR)-based (targeting known mutations) or next-generation sequencing (NGS)-based methods for a broader mutational spectrum. PCR approaches are faster, cheaper, and require known mutational status, while NGS provides greater depth [17,131,132].

As described earlier, CoM shares many genetic features with cutaneous melanoma, including mutations in the BRAF, NRAS, NF1, and c-KIT genes [6,119]. Tian expanded this panel to include BRAF, NRAS, KRAS, NF1, NF2, GNAQ, GNA11, retinoblastoma 1 (RB1), phosphatase and tensin homolog (PTEN), tumour protein p53 (TP53), AKT serine/threonine kinase (AKT1/2), Breast Cancer Susceptibility Gene (BRCA1/2), cyclin-dependent kinase inhibitor 2A (CDKN2A), catenin beta 1 (CTNNB1), and Neurogenic Locus Notch Homolog (NOTCH1/2), showing that plasma ctDNA positivity correlated with distant but not regional metastasis [86].

UM has been more intensively investigated; contemporary studies increasingly use tumour-informed ctDNA assays that target patient-specific drivers (e.g., GNAQ, GNA11, BAP1), thereby enhancing MRD-detection sensitivity. GNAQ and GNA11 mutations occur in ~80% of cases [23], and Metz detected ctDNA for these mutations in 41% of patients using PCR [133]. Most UM ctDNA studies utilise real-time PCR (RT-PCR) for tyrosinase, melanoma antigen recognised by T cells 1 (MELAN-A/MART-1) and gp100 transcripts [120,134,135]. Schuster reported a 60% positivity rate in metastatic UM and an association with disease progression [134,135]. Keilholz reported significant increases in marker positivity between primary and metastatic UM: tyrosinase, from 12.5% to 60%; MELAN-A, from 4% to 77%; and MART1, from 4% to 10% [136].

Epigenetic regulation contributes additional variability. Loss of BAP-1 expression in tumour tissue correlates with metastatic risk and may be detectable in liquid biopsy [137]. Key limitations include detection challenges in rare mutation subtypes and a lack of standardised thresholds for ctDNA quantification across disease stages.

### 5.4. Minimal Residual Disease Detection

MRD is a pivotal predictor of long-term outcome in ocular melanoma, marking microscopic tumour deposits that survive local therapy. Standard imaging—ultrasound, OCT, MRI, and routine surveillance—lacks the sensitivity to detect these early micrometastases, often resulting in delayed recognition of metastatic spread [1,8,13,14,15]. Importantly, radiologic techniques consistently miss MRD, now recognised as a major driver of late metastatic relapse across solid tumours [16,17].

In UM, MRD hinders survival improvement; disseminated cells can remain dormant and undetectable for years before causing hepatic metastases, accounting for ~50% of cumulative metastatic incidence [16,17]. The inability of conventional surveillance methods to identify microscopic dissemination highlights the urgent need for more sensitive and dynamic biomarkers.

Liquid biopsy technologies, particularly ctDNA, have shown promise in addressing this diagnostic gap. ctDNA enables the detection of tumour-derived genetic alterations at extremely low variant allele fractions, allowing for earlier identification of molecular relapse before radiographic progression [16,17]. Recent studies demonstrate that ctDNA-based MRD assays can not only detect subclinical metastatic activity but also track clonal evolution, offering insights into tumour dynamics and mechanisms of therapeutic resistance [18,19]. In UM, this approach has shown particular relevance for high-risk molecular subtypes, such as BAP1-mutant tumours, where early molecular progression may precede detectable radiologic changes.

Although data for CoM remain limited, early investigations suggest that MRD-oriented ctDNA profiling could improve prognostication, guide systemic therapeutic decision-making, and facilitate risk-adapted surveillance strategies [19]. As assay sensitivity improves and multi-omic integration advances, MRD detection is expected to become a central component of precision follow-up in both UM and CoM.

Overall, MRD-focused liquid biopsy represents a pivotal step toward earlier metastasis detection, more personalised surveillance, and biomarker-guided therapeutic intervention in ocular melanoma. Continued development and standardisation of ctDNA-based MRD assays, alongside extensive multicentre validation efforts, will be essential for their full clinical integration in ocular oncology [18,19].

### 5.5. Exosomes and Circulating miRNAs

Exosomal miRNAs are promising circulating biomarkers for stratifying metastasis risk, although analytical sensitivity and specificity vary across platforms [116,132,138,139]. They also modulate metastatic niche formation and tumour microenvironment remodelling, and can serve as therapeutic delivery vectors that cross the blood–brain barrier [140,141,142].

UM demonstrates bimodal metastatic behaviour, with an early disseminated but clinically silent phase followed by reactivation [142]. Exosomal integrins contribute to metastatic awakening and organotropism. miRNAs transferred via exosomes enhance metastatic potential, tumour proliferation, angiogenesis, and reprogramming of fibroblasts and macrophages toward a tumour-supportive phenotype [34,143,144,145]. This real-time messaging makes exosomal miRNAs powerful, dynamic biomarkers, although a lack of standardisation limits their clinical use [132].

Proteomic profiling further expands their utility. Surman identified 66 cancer-related proteins in UM exosomes, including heat-shock protein β-1, which is differentially expressed between primary and metastatic disease [144]. Pessuti reported distinct proteomic signatures in intra-ocular fluids versus plasma: aqueous/vitreous samples are enriched for anti-apoptotic and angiogenic proteins, whereas plasma contains proteins that regulate metastasis [143]. Primary UM cell lines secrete organ-tropism proteins, while metastatic lines release extracellular matrix remodellers, including MMPs, collagens, and nidogen-1. Correspondingly, ergoline exposure down-regulated metastasis-associated proteins in OMM2.5-derived exosomes [123].

### 5.6. Comparative Clinical Perspectives in UM and CoM

In the UM settings, liquid biopsy demonstrated high clinical value for prognostication and surveillance. miRNAs distinguish UM from benign melanocytic lesions; CTCs enable real-time metastatic risk stratification; and ctDNA reflects tumour burden, metastatic evolution, and treatment response well before radiological findings. Sequencing of ctDNA may detect emerging resistance mutations and inform immunotherapy selection [16,19,116].

In CoM, clinical utility remains limited; however, emerging research into exosomal and circulating miRNA biomarkers may change this landscape. Since histological biopsy is routinely performed for diagnosis and mutation testing, interest in liquid biopsy has been relatively low to date. Current research focuses primarily on early detection of metastasis, molecular profiling, and personalised treatment strategies [3,86].

Both UM and CoM may benefit from exosome-based biomarkers and the potential use of exosomes as therapeutic carriers; however, translating these findings into clinical practice remains in its early stages.

## 6. Clinical and Translational Applications

Liquid biopsy approaches are best viewed as complementary to conventional imaging modalities, providing additional molecular information rather than replacing radiologic surveillance. Traditional imaging and histopathology remain essential, but do not adequately capture tumour heterogeneity, clonal evolution, or minimal residual disease. Advances in genomics, transcriptomics, and circulating biomarkers now enable the earlier detection of metastasis, refined risk prediction, and personalised systemic therapy [120,146]. Consequently, both UM and CoM are moving from standardised treatment regimens toward precision oncology, where clinical decisions are guided by measurable molecular characteristics rather than tumour size or location alone [2,3,127]. Ocular melanoma is particularly well suited to liquid biopsy because metastatic spread occurs predominantly in the liver, surgical biopsy of hepatic lesions is often difficult or unsafe, and patients require long-term surveillance over many years, making repeated non-invasive sampling clinically advantageous [11,40,86].

### 6.1. Molecular-Based Risk Stratification

Prognostic classification in UM has been transformed by GEP and chromosomal analysis, which stratify tumours into Class 1 (low risk) and Class 2 (high risk) [25,56]. Class 2 profiles strongly correlate with *BAP1* loss, monosomy 3, and a high probability of hepatic metastasis within five years [14]. However, radiologic surveillance with ultrasound, MRI, or CT frequently detects metastases only after substantial tumour burden has developed, limiting therapeutic options [64].

Liquid biopsy adds a dynamic layer of surveillance by enabling molecular monitoring over time. ctDNA, CTCs, and exosome-derived miRNAs correlate with tumour activity and metastatic spread [17]. In high-risk patients, rising ctDNA levels may prompt intensified liver imaging, referral for clinical trials, or initiation of systemic therapy before imaging becomes positive [16]. Conversely, persistently negative ctDNA could justify lengthening imaging intervals, lowering cost and radiation exposure without compromising safety [127].

Evidence supporting similar approaches in CoM is emerging. Although its mutation spectrum differs from UM, ctDNA and exosomal miRNAs have been detected in both localised and metastatic CoM. Their ability to identify early recurrence is particularly relevant in younger patients, in whom cumulative radiation exposure from repeated imaging is a long-term concern [86,99].

### 6.2. Biomarker-Guided Surveillance Strategies

The integration of molecular and circulating biomarkers into clinical workflows has enabled the development of risk-adapted surveillance strategies tailored to tumour biology rather than anatomical features alone. These approaches are particularly relevant in ocular melanoma, where early detection of metastatic spread remains a major clinical challenge [3,16,17,19,40,41,42].

#### 6.2.1. Uveal Melanoma

UM remains challenging to treat systemically because its driver mutations, *GNAQ* and *GNA11*, are not currently druggable. Instead, secondary alterations carry therapeutic significance. Loss of *BAP1* is associated with aggressive tumour behaviour, immune suppression, and chromosomal instability, features that shape surveillance and systemic therapy selection [27,137].

Tebentafusp represents the first systemic treatment to demonstrate an overall survival benefit in metastatic UM. Its activity depends on the human leukocyte antigen (HLA)-A*02:01-restricted presentation of gp100, making molecular screening essential for determining eligibility [14,59,147]. Importantly, reductions in ctDNA after initiation of tebentafusp correlate strongly with survival, even in the absence of a radiologic response, establishing ctDNA as a valuable pharmacodynamic biomarker [17].

#### 6.2.2. Conjunctival Melanoma

The molecular profile of CoM parallels that of cutaneous melanoma, enabling translation of targeted and immune-based therapies. Approximately one-third of CoMs harbour BRAF V600E mutations. Case series report regression of metastases and durable responses with combined BRAF and MEK inhibition, supporting the routine mutation testing in advanced disease [2,21].

NRAS-mutant tumours activate MAPK signalling independently of BRAF and are therefore resistant to BRAF inhibitors. However, MEK inhibitors, whether used alone or in combination with immunotherapy, have shown encouraging early-phase activity. NF1-mutated tumours often display high TMB and increased neoantigen load, providing a biological rationale for first-line checkpoint inhibition. Although less frequent, C-KIT mutations create opportunities for targeted therapy with tyrosine kinase inhibitors such as imatinib or dasatinib. Responses are typically partial and time-limited, but clinically meaningful in certain cases [28,72,73].

Collectively, these findings demonstrate that metastatic CoM is a heterogeneous disease composed of molecularly distinct subgroups. Routine genomic profiling is therefore a critical component of personalised systemic therapy.

### 6.3. Circulating Tumour DNA and RNA

In UM, clinical pathways increasingly begin with molecular classification. Class 1 tumours typically undergo imaging at 6–12-month intervals, with liquid biopsy reserved for specific scenarios. Class 2 tumours typically undergo more intensive surveillance, often at 3–4-month intervals, with increasing interest in the adjunctive use of serial ctDNA trend analysis. Rising ctDNA levels in the absence of radiologic progression may justify enhanced liver imaging or early enrolment into systemic therapy trials. For HLA-A*02:01-positive patients with metastatic disease, tebentafusp is a rational first-line option, and ongoing ctDNA monitoring can guide continuation or escalation of therapy [64,148,149].

In CoM, systemic therapy selection is driven by biomarker status [28,60,73].

- BRAF-mutant disease → BRAF + MEK inhibitors;
- NRAS-mutant disease → MEK inhibition ± immunotherapy;
- NF1-mutant disease → checkpoint blockade;
- C-KIT-altered disease → tyrosine kinase inhibitors.

As liquid biopsy platforms mature, ctDNA and exosomal miRNA signatures may guide the early detection of relapse and real-time monitoring of therapeutic response, supporting more adaptive treatment strategies [19,86].

### 6.4. Barriers to Clinical Translation

Despite clear clinical potential, multiple challenges limit widespread implementation.

Technical barriers include low ctDNA concentrations in early-stage disease, variability in exosome isolation methods, and lack of standardised thresholds for biomarker positivity across laboratories [33,117,122,150].

Regulatory barriers persist because many assays remain investigational and are not approved for routine clinical use. Prospective validation is challenging due to the rarity of ocular melanoma and the limited availability of large patient cohorts [38,40]. Economic barriers include high sequencing costs, reimbursement uncertainty, and restricted access to molecular diagnostics outside specialised centres [151].

Addressing these obstacles will require multi-institutional collaboration, standardised assay methods, harmonised reporting criteria, and biomarker-stratified clinical trials.

### 6.5. Future Directions in Biomarker-Guided Patient Care

Precision oncology in ocular melanoma is evolving toward the integration of a multi-omics approach. Combining genomics, transcriptomics, proteomics, radiomics, and immune profiling may improve the prediction of metastatic behaviour and treatment response. Machine learning models applied to ctDNA kinetics, imaging data, and tumour microenvironment markers could refine surveillance schedules and identify treatment failures earlier than imaging alone and are moving toward multi-omics integration [16,68,152].

Table 4 summarises clinically actionable biomarkers and their corresponding systemic treatment pathways in ocular melanoma, emphasising the potential of liquid biopsy for real-time therapeutic monitoring [14,16,25,33,40,41,58,59,60,65,67,118,135,136,138,153,154,155,156].

## 7. Challenges and Limitations

The rapid evolution of molecular profiling and liquid biopsy technologies is reshaping the clinical management of ocular melanoma. Future research efforts must focus on translating these advances into standardised, clinically validated tools that enable truly personalised surveillance and treatment [2,3,16,17,19,41,120].

### 7.1. Epidemiological Rarity and Limited Cohort Size

UM and CoM remain among the rarest solid malignancies, with incidence rates of approximately 5–7 cases per million for UM and 0.2–0.5 per million for CoM [2,3,7,13,20,157,158]. As a consequence, most molecular and translational studies are based on small, single-centre or retrospective cohorts, which limit statistical power, hinder robust multivariable modelling, and increase the risk of overfitting, particularly in multi-omics datasets and machine-learning-based prediction models. These constraints reduce external validity and complicate the development of clinically reliable biomarkers for metastasis risk stratification or therapeutic response. This scarcity also results in heterogeneous patient populations and a reliance on retrospective case series, making it challenging to distinguish accurate biological signals from centre-specific artefacts and contributing to inconsistent biomarker performance across studies [3,21,25,158].

Access to tumour tissue is another challenge. In UM, intraocular biopsies are performed infrequently due to the risks of tumour seeding and vision-threatening complications, which restrict opportunities for routine genomic profiling [4,14,121]. In CoM, although there is overlap with cutaneous melanoma driver mutations, the rarity of the disease limits the prospective validation of targeted therapies and biomarker-guided treatment pathways [28,73].

### 7.2. Standardisation and Reproducibility Challenges

The fragmented nature of research on ocular melanoma is exacerbated by the lack of universal protocols for tissue acquisition, processing, and molecular analysis, resulting in significant inter-laboratory variability in biomarker results [62]. Substantial methodological heterogeneity exists across tissue-based and liquid-biopsy platforms. ctDNA in UM may be detected using digital droplet PCR, BEAMing, or ultra-deep next-generation sequencing, each with distinct analytical sensitivities (limit of detection ~0.01–0.1%), variant-calling pipelines, and quality-control thresholds [17,33,116,159]. Likewise, exosome isolation methods (ultracentrifugation, size-exclusion chromatography, immunocapture) and CTC enrichment technologies (immunomagnetic capture vs. microfluidics) vary widely between laboratories [120,127,160]. Downstream microRNA profiling also lacks consensus analytical protocols, leading to divergent results and limiting reproducibility between centres [101,103,161].

False-negative liquid biopsy results represent an additional methodological challenge. Despite promising clinical utility, both CTC and ctDNA analyses may yield false-negative outcomes, particularly in patients with low tumour burden or micrometastatic disease below the detection threshold. Technical variation in capture efficiency, short DNA-fragment half-lives, and heterogeneous biomarker expression further contribute to underdetection. These limitations underscore the need for multi-marker panels, longitudinal sampling, and standardised analytical pipelines [17,151,162,163,164]. The development of highly sensitive assays, such as digital droplet PCR, for detecting ctDNA has shown promise in improving the detection of UM driver mutations in patient blood and aqueous humour, thereby enhancing diagnostic capabilities for this often-asymptomatic presenting cancer [17,117].

The absence of universally accepted pre-analytical standard operating procedures, including blood collection tubes, plasma processing times, storage conditions, and RNA stabilisation, leads to poor inter-laboratory reproducibility and complicates cross-study comparison [19,162,165]. Without standardised analytical quality-control systems, biomarker signals may be artefactual rather than disease-driven, further delaying clinical acceptance [162,165]. Without large, prospective, multicentre validation trials, most candidate biomarkers remain confined to experimental research settings rather than being incorporated into clinical guidelines [18,19].

### 7.3. Clinical, Economic, and Regulatory Barriers

Even when biomarkers demonstrate diagnostic or prognostic utility, their clinical implementation is hindered by cost, limited access, and regulatory pathways. High-throughput multi-omics platforms, liquid biopsy assays, and bioinformatics pipelines require specialised infrastructure and training, and are not routinely reimbursed in most healthcare systems [18,25]. Whole-genome sequencing, methylation arrays, and proteomic platforms remain financially inaccessible for many ophthalmic oncology centres, particularly those without dedicated molecular pathology units [19,25,166].

Economic and logistical considerations further limit translation into routine care. High-throughput sequencing platforms, digital PCR, and exosome isolation technologies remain costly and unevenly distributed across centres. The absence of standardised assays increases analytical variability and raises barriers for routine reimbursement. Broad implementation will require cost-effective workflows, external quality control programmes, and multicentre harmonisation [152,167].

Although ctDNA can detect progressive or residual disease in UM, it is rarely used in routine surveillance because no approved clinical algorithms define when ctDNA changes should trigger imaging, treatment modification, or referral to oncology [17,40].

Similarly, while BRAF or KIT mutations in CoM may guide targeted therapy with BRAF/MEK inhibitors, real-world application remains inconsistent due to variability in drug availability, cost, and companion diagnostic approval [3,19,21,168]. The regulatory landscape for ocular oncology-specific molecular diagnostics lags behind that of cutaneous melanoma, discouraging adoption outside research centres.

Ethical and legal considerations further complicate the biomarker research. Biobanking of ocular tumour tissue and liquid biopsy samples requires harmonised consent procedures, anonymisation standards, and compliance with data protection regulations such as the GDPR, which can delay multi-country collaborations and data sharing. Regulatory bodies, such as the FDA and European Medicines Agency (EMA), have yet to approve most liquid biopsy panels for routine clinical use in UM, creating a significant hurdle to widespread adoption despite increasing evidence of their diagnostic and prognostic value [40,116]. This regulatory gap often necessitates the use of these advanced molecular tools as "laboratory-developed tests", which, while permissible, typically lack the robust validation and oversight associated with FDA-approved diagnostics, further hindering their widespread clinical integration and reimbursement [120].

### 7.4. Biologic and Clinical Heterogeneity

Both UM and CoM exhibit intratumoural heterogeneity and subclonal evolution, meaning that a single tissue biopsy may fail to capture the full range of actionable mutations [2]. Liquid biopsy partially addresses this limitation by detecting tumour-derived material released into circulation; however, sensitivity remains suboptimal in early-stage disease or micrometastatic UM, particularly in the liver, where tumour burden may fall below assay detection thresholds. Early post-treatment surveillance is particularly affected, as circulating biomarkers may be undetectable despite the biologically relevant residual disease [17,19,86,116,117,169,170]. ctDNA is detectable in the plasma of a significant proportion of metastatic UM patients, often preceding clinical detection of metastases by several months [25].

Biomechanical and physiological barriers also contribute to low detection rates. The blood–ocular barrier restricts the passive release of tumour-derived cells and nucleic acids into the systemic circulation, thereby reducing the availability of biomarkers compared with other solid tumours [17,25,117,120,170]. Differences in vascularisation between uveal and conjunctival melanoma further influence detectability, and advanced isolation platforms with improved sensitivity may be required to overcome these anatomical constraints. Additional research is needed to refine these technologies and validate their clinical utility across diverse patient cohorts, particularly those with early-stage disease [2,17,120].

Exosomal miRNA and CTC biomarkers are also influenced by systemic inflammation, tumour necrosis, and therapeutic pressure, complicating the interpretation of longitudinal changes in these biomarkers [34,127].

### 7.5. Limitations of Artificial Intelligence and Data Integration

Artificial intelligence (AI) and machine learning (ML) offer promising tools for integrating genomic, transcriptomic, proteomic, and imaging data. Still, most published models are derived from small, institution-specific cohorts and lack adequate external validation, limiting their generalizability and predictive accuracy [25,152]. This increases the risk of overfitting and limits clinical applicability. Without harmonised multicentre datasets and regulatory-grade analytical standards, AI-based biomarker discovery cannot progress to decision-support algorithms suitable for routine care. The absence of curated, standardised ocular melanoma biobanks with linked clinical outcomes further restricts the development of predictive AI models [25,171]. Collaborative data-sharing initiatives, such as The Collaborative Ocular Melanoma Study, are crucial for assembling comprehensive UM patient databases that support robust AI model development and validation [25].

Together, these limitations emphasise the need for international consortia, harmonised Standard Operating Procedures (SOPs), dedicated biobanking infrastructure, and prospective trials with clearly defined clinical endpoints. Only through sufficiently powered multicentre validation can emerging molecular and liquid biopsy biomarkers transition from exploratory research to clinically actionable, reimbursable tools. This integrated approach is crucial for overcoming current challenges in the diagnosis, prognosis, and monitoring of ocular melanoma treatment, ultimately improving patient outcomes [172].

The limitations of existing approaches simultaneously drive the development of advanced multi-omics models, standardised international biobanks, and innovative liquid biopsy techniques, creating opportunities for new diagnostic paradigms, earlier disease detection, and personalised therapeutic strategies that could significantly improve outcomes in ocular oncology [152,173].

## 8. Future Perspectives

Integrating genomic, epigenomic, and liquid biopsy platforms is expected to fundamentally reshape risk stratification, surveillance, and therapeutic decision-making in ocular melanoma [2,3,4,6,14,17,19,24,25,33,40,41,60,63,111,116].

### 8.1. Multi-Omics Integration and Systems Oncology

Future research will increasingly shift from static, single-parameter biomarkers toward integrated, dynamic profiling across multiple molecular layers. Although traditional UM prognosis relied on GNAQ/GNA11 mutations, BAP1 loss, and GEP classes, emerging work shows that combining mutations, copy-number changes, methylation signatures, transcriptomic programmes, proteomic pathways, and metabolomic data offers substantially greater predictive resolution [16,17,25,45,46,51,57,61,66,70]. Integrated multi-omic analysis provides a deeper understanding of tumour biology and therapeutic resistance.

Dynamic assessment of these molecular layers through liquid biopsies may allow real-time monitoring of metastatic evolution and treatment response [25]. Preferentially expressed antigen in melanoma (PRAME) expression improves the prognostic accuracy of 15-GEP and enhances risk classification in primary UM [56]. Proteomic studies have identified biomarkers, such as CDH1 and HLA-DPA1, as well as signalling kinases with potential relevance for immune checkpoint strategies [25].

As tumour atlases expand, risk assessment is expected to transition from categorical staging to continuous, updateable probability curves. This will enable fine-grained identification of high-risk patients, guiding intensified surveillance and selective use of adjuvant therapies—critical in UM, where metastatic treatment options remain limited [9,62]. Multi-omics integration may also clarify mechanisms of tumorigenesis, immune escape, and treatment resistance, supporting more personalised intervention strategies [25].

### 8.2. Liquid Biopsy as a Central Tool in Clinical Practice

A major unmet need in ocular melanoma is the lack of minimally invasive, sensitive biomarkers for early detection of metastasis. Tissue biopsies are informative but limited by invasiveness and sampling bias [33]. Liquid biopsy modalities, including ctDNA, CTCs, exosomes, and miRNAs, enable repeated sampling and real-time molecular profiling, thereby improving monitoring of disease progression, recurrence, and therapeutic response [25,172].

ctDNA harbouring GNAQ/GNA11 mutations can predict metastatic progression months before radiographic detection [25]. Blood-based biomarkers can detect MRD after primary therapy, and when surgical biopsies are unsafe, ctDNA and CTCs provide actionable molecular information, including BRAF and NRAS mutations relevant for targeted therapy [19].

Broad clinical adoption will require standardisation of pre-analytical workflows, reference materials, and detection thresholds. Once harmonised, liquid biopsy endpoints are likely to be incorporated into biomarker-driven clinical trials as surrogate predictors of response or progression [18,25,33,167].

### 8.3. Artificial Intelligence and Computational Modelling

As datasets become increasingly complex, AI and machine learning will become essential for clinical translation. Algorithms such as random forests and gradient-boosted trees can incorporate hundreds of genomic, transcriptomic, and proteomic variables to predict metastasis risk [152,171,174]. Deep-learning approaches applied to transcriptomic or methylation data may identify new molecular subtypes responsive to immunotherapy or epigenetic agents.

AI methods can also improve liquid biopsy interpretation by distinguishing tumour-derived ctDNA based on fragmentation patterns and methylation signatures [153,175]. These advancements enhance MRD detection and monitoring [25]. Over time, AI-guided models may be able to predict relapse trajectories, enabling earlier intervention [176].

### 8.4. Remaining Biological Gaps

Important biological questions remain unresolved. Therapeutic targeting of GNAQ/GNA11 signalling remains challenging despite its early driver role [23]. Agents targeting PKC, YAP/TEAD, or ARF signalling have shown limited progress toward pivotal trials [22,23]. SF3B1-mutant tumours represent a unique intermediate-risk subgroup; spliceosome inhibitors such as H3B-8800 may have potential [177,178].

BAP1 loss remains a key determinant of immune evasion, and an immunologically “cold” tumour microenvironment requires innovative combination strategies, such as tebentafusp plus PD-1 blockade [57,179].

### 8.5. Technical and Clinical Gaps

Standardisation of pre-analytical workflows and assay sensitivity is essential, particularly for low-abundance ctDNA or CTCs [17]. Robust clinical validity has been established for several tissue-based biomarkers in uveal melanoma, including GEP classification, BAP1 loss, monosomy 3, and chromosome 8q gain, which are already integrated into routine risk stratification. In contrast, most liquid biopsy biomarkers, such as ctDNA, CTCs, and circulating or exosomal miRNAs, are currently supported predominantly by preclinical studies and small retrospective or single-centre cohorts.

Despite strong translational promise, several technological and regulatory barriers continue to limit the routine clinical implementation of liquid biopsy approaches in ocular melanoma. Key technological challenges include the lack of standardised pre-analytical and analytical workflows (sample collection, processing, assay platforms, and reporting thresholds), low biomarker abundance in early-stage disease, partly attributable to the blood–ocular barrier, and substantial inter-laboratory variability in ctDNA, CTC, and microRNA detection methods. Regulatory barriers include the absence of FDA- or EMA-approved ocular melanoma-specific liquid biopsy assays, limited reimbursement pathways, and reliance on laboratory-developed tests without harmonised quality control frameworks. Together, these limitations highlight the need for standardised assay development, biomarker-driven prospective clinical trials, and multi-institutional collaboration to achieve clinical-grade validation and enable the integration of liquid biopsy biomarkers into routine precision oncology care for ocular melanoma.

Tumour heterogeneity requires spatially resolved multi-omics, while large-scale datasets demand robust bioinformatic pipelines [25]. Prospective studies integrating longitudinal liquid biopsy data with genomic and clinical information remain limited, especially given differences between UM and cutaneous melanoma [17,62]. Current biomarkers cannot precisely predict the timing of metastasis, underscoring the need for temporally accurate prognostic tools [25]. Highly sensitive ctDNA methods show promise for MRD detection, though mechanisms of ctDNA release remain unclear [131,172]. Further progress requires global registries, harmonised biospecimen collection and diverse cohorts to address ancestry-related gaps [4,25,180].

### 8.6. Strategic Roadmap for Clinical Integration

A strategic translational roadmap for ocular melanoma is emerging. Within this evolving framework, the clinical validity and translational readiness of currently available biomarkers remain heterogeneous and should be interpreted in relation to their intended clinical application. Tissue-based biomarkers in UM, including BAP1 status, monosomy 3, chromosome 8q gain, and gene expression profiling, represent the most clinically mature tools, supported by prospective validation studies and already incorporated into routine risk stratification and surveillance strategies.

In contrast, most liquid biopsy approaches, such as ctDNA, CTCs, and circulating or exosomal miRNAs, are currently positioned earlier along the translational pathway. While multiple studies demonstrate meaningful associations with tumour burden, metastatic progression, and treatment response, existing evidence is derived predominantly from preclinical models, small retrospective cohorts, and exploratory translational studies. The current lack of clinical-grade validation and harmonised implementation frameworks continues to limit their routine clinical adoption, positioning these biomarkers as promising but still investigational tools within the strategic roadmap.

For CoM, translational progress is further constrained by lower disease incidence and biological heterogeneity. Molecular insights have largely been extrapolated from cutaneous melanoma, while robust, disease-specific biomarker validation remains limited. Nevertheless, emerging genomic, immune-related, and liquid biopsy biomarkers may support improved risk stratification and therapeutic monitoring as larger collaborative cohorts and prospective studies become available.

Clustered regularly interspaced short palindromic repeats (CRISPR)-engineered UM organoids that recapitulate key genetic alterations, such as GNAQ/GNA11, BAP1, SF3B1, or EIF1AX, can be used to screen combinatorial drug regimens and predict mechanisms of response or resistance [46]. Adaptive Phase I/II clinical trial platforms, guided by liquid biopsy endpoints, provide a rapid pathway for testing novel agents and incorporating biomarker-defined patient subsets, eliminating the delays associated with traditional trial redesign [18]. Parallel efforts to link national cancer registries with liquid biopsy laboratories will generate real-world evidence, enabling longitudinal analysis of ctDNA kinetics, therapeutic exposure, and survival outcomes [4,25,180]. These coordinated developments represent essential steps toward a future in which treatment decisions are informed by dynamic biological data rather than static clinical impressions.

**Figure 2:**
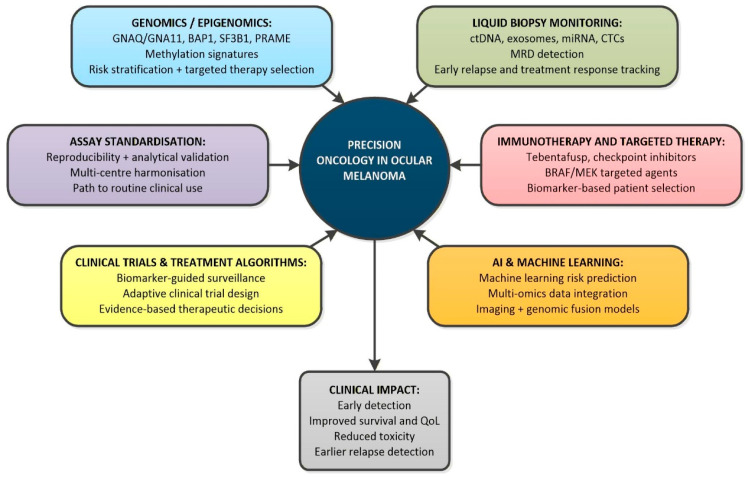
Integration of tissue-based and liquid biopsy biomarkers in ocular melanoma. A conceptual model illustrating how genomic, epigenetic, proteomic, and circulating biomarkers converge to guide risk stratification, surveillance, and selection of systemic therapies. GNAQ/GNA11: BAP1: BRCA1-associated protein 1; SF3B1: splicing factor 3b subunit 1; PRAME: preferentially expressed antigen of melanoma; ctDNA: circulating tumour DNA; miRNA: microRNA; CTCs: circulating tumour cells; MRD: minimal residual disease; BRAF/MEK: AI: artificial intelligence; QoL: quality of life.

Figure 2 presents a conceptual overview of integrating molecular and liquid biopsy biomarkers into precision oncology workflows for ocular melanoma [3,14,25,40,64,83,118,121,151,157,163,164,165,171,172,175,176]. The combination of integrated multi-omics, liquid biopsy innovations, and AI-driven analytics reflects a paradigm shift toward continuous monitoring, early intervention, and personalised therapy, with the potential to improve clinical outcomes for patients with ocular melanoma [17,25,116].

## 9. Conclusions and Future Research Directions

Ocular melanoma represents a biologically heterogeneous group of malignancies in which advances in molecular profiling and liquid biopsy technologies are rapidly reshaping clinical management. By integrating genomic, epigenetic, transcriptomic, proteomic, and circulating biomarkers, precision oncology approaches are increasingly moving beyond static risk stratification toward dynamic, biology-driven surveillance and treatment strategies.

In UM, tumour behaviour is primarily determined by secondary genetic and epigenetic alterations rather than by early driver mutations, whereas CoM shares key molecular features with cutaneous melanoma, allowing for the application of targeted and immune-based therapeutic approaches. These fundamental biological differences underscore the need for tailored biomarker strategies rather than uniform models across ocular melanoma subtypes. This review highlights that no single biomarker is sufficient to capture the biological complexity, heterogeneity, and metastatic potential of ocular melanoma. Instead, integrating tissue-based molecular profiling with dynamic liquid biopsy approaches provides a more comprehensive and clinically meaningful assessment of tumour risk and evolution.

Liquid biopsy biomarkers, particularly ctDNA and exosome-derived microRNAs, hold considerable promise for the detection of minimal residual disease, longitudinal monitoring of treatment response, and earlier identification of metastatic progression—often preceding radiologic detection. When incorporated into risk-adapted surveillance strategies, these tools may enable more timely therapeutic intervention and improved patient stratification for clinical trials.

Despite this potential, widespread clinical implementation remains constrained by technical heterogeneity, limited standardisation, and the rarity of ocular melanoma, which hampers large-scale prospective validation. Future progress will depend on multi-institutional collaboration, harmonised assay platforms, and biomarker-driven clinical trial designs that incorporate molecular and circulating biomarkers as actionable endpoints.

In conclusion, the convergence of genomics, epigenetics, proteomics, and liquid biopsy technologies represents a critical step toward truly personalised precision oncology in ocular melanoma. By bridging molecular discovery with clinical application, integrated biomarker strategies have the potential to transform surveillance paradigms, optimise therapeutic decision-making, and ultimately improve outcomes for patients with both uveal and conjunctival melanoma.

## Figures and Tables

**Table 1:** Molecular biomarkers in uveal melanoma: functional roles, prognostic significance, and therapeutic potential.

Biomarker/Pathway	Frequency	Molecular/Functional Role	Prognostic Value	Therapeutic/Translational Implications
*GNAQ*	45–55%	Activates Gαq–PKC–MAPK Initiates tumorigenesis	Early driver mutation Not predictive of metastasis	PKC, MEK inhibitors (under study)
*GNA11*	30–40%	Activates Gαq–PKC–MAPK Mutually exclusive with *GNAQ*	Higher metastatic potential than *GNAQ*	Gαq/11-Tri-complex inhibitors (preclinical)
CYSLTR2 p.L129Q	2–4%	Constitutive GPCR activation → GNAQ-like signalling Mutually exclusive with *GNAQ/GNA11*	Rare driver Intermediate risk	Targetable GPCR mutation (preclinical)
*PLCB4/PLCB4* mutations	3–4%	Activates PKC downstream of Gαq; Mutually exclusive with *GNAQ/GNA11*	Uncommon Moderate risk	Potential sensitivity to PKC inhibitors
*BAP1* (loss/inactivation)	40–45%	Tumour suppressor Chromatin regulation DNA repair	Strongest predictor of metastasis Poor survival	Stratify for intensive surveillance Epigenetic therapy trials
*SF3B1*	20–25%	Spliceosome mutation → aberrant RNA splicing	Late-onset metastasis Intermediate risk	Splicing-targeted therapy trials
*EIF1AX*	8–19%	Translation initiation factor	Favourable prognosis Low metastatic risk	Routine surveillance No targeted therapies
*SRSF2*	4–6%	Alternative splicing disruption	Intermediate risk	Experimental spliceosome inhibitors
Monosomy 3	45–50%	Loss of one chromosome 3 copy Linked to *BAP1* loss	Highest metastatic risk Class 2 phenotype	Guides intensive surveillance, immunotherapy relevance
Chromosome 8q Gain	40–55%	Increased oncogenic dosage (e.g., *MYC*) chromosomal instability	Amplifies metastatic risk, especially with monosomy 3	High-risk classification biomarker for clinical trial selection

BAP1, BRCA1-associated protein 1; EIF1AX, eukaryotic translation initiation factor 1A, X-linked; Gαq, G protein alpha q subunit; GNA11, G protein subunit alpha 11; GNAQ, G protein subunit alpha q; CYSLTR2: cysteinyl leukotriene receptor 2; GPCR, G protein–coupled receptor; MAPK, mitogen-activated protein kinase; MEK, MAPK/ERK kinase; MYC, MYC proto-oncogene, bHLH transcription factor; PKC, protein kinase C; PLCB4, phospholipase C beta 4; SF3B1, splicing factor 3b subunit 1; SRSF2, serine/arginine-rich splicing factor 2.

**Table 2:** Molecular biomarkers in conjunctival melanoma: functional roles, prognostic significance, and therapeutic implications.

Biomarker/Pathway	Frequency	Molecular/Functional Role	Prognostic Significance	Therapeutic/Translational Implications
*BRAF* V600E	30–50%	Constitutive MAPK activation	Aggressive behaviour in some cohorts	BRAF + MEK inhibitors Clinically effective in case reports
*NRAS*	15–25%	MAPK activation independent of *BRAF* Mutually exclusive with *BRAF*	Poor prognosis UV-associated tumours	MEK inhibitors ± immunotherapy Limited clinical data
*NF1* Loss	33–50%	Negative regulator of *RAS* Hyperactivated MAPK	High mutational burden Potential immune responsiveness	Immune checkpoint inhibitors MAPK inhibitors
*PTEN* Loss/Mutation	4–14%	Activates PI3K–AKT–mTOR	Worse prognosis High metastatic risk Resistance to MAPK therapy	mTOR inhibitors (targeted therapy candidates)
*PIK3CA*	<3%	Upstream PI3K activation	Moderate risk	PI3K inhibitors (preclinical)
*KIT* (mutation/amplification)	2–7%	Activates MAPK + PI3K	Variable prognosis	KIT-targeted therapies (Imatinib, Dasatinib; clinical activity in subset)
*MET* Overexpression	<5%	Promotes metastasis and invasiveness	Metastatic progression	MET inhibitors (exploratory) Potential KIT-targeted therapy

AKT, protein kinase B; BRAF, B-Raf proto-oncogene, serine/threonine kinase; KIT, KIT proto-oncogene, receptor tyrosine kinase; MAPK, mitogen-activated protein kinase; MEK, MAPK/ERK kinase; MET, MET proto-oncogene, receptor tyrosine kinase; mTOR, mechanistic target of rapamycin; NF1, neurofibromin 1; NRAS, NRAS proto-oncogene, GTPase; PI3K, phosphoinositide 3-kinase; PIK3CA, phosphatidylinositol-4,5-bisphosphate 3-kinase catalytic subunit alpha; PTEN, phosphatase and tensin homolog; RAS, rat sarcoma viral oncogene homolog; UV, ultraviolet.

**Table 3:** Epigenetic, transcriptomic, and protein biomarkers in ocular melanoma: functional roles, prognostic significance, and therapeutic potential.

Biomarker Class	Representative Biomarkers	Molecular/Functional Role	Prognostic/Diagnostic Significance	Therapeutic/Translational Potential
DNA Methylation	BAP1, SF3B1, APC, RASSF1A, CDKN2A	Epigenetic silencing of tumour suppressor genes	Hypermethylation predicts metastasis Distinguishes benign from malignant lesions	Candidate for non-invasive ctDNA methylation assays
RNA Methylation (m^6^A)	METTL3, ALKBH5, FTO, HINT2	Modulates RNA stability and translation Promotes angiogenesis and proliferation	Low METTL3/high ALKBH5 associated with poor outcome	Targetable with epitranscriptomic modulators
miRNAs	↑ miR-21, miR-146b, let-7b, miR-199a; ↓ miR-204, miR-211	Regulate EMT, angiogenesis, and immune evasion	Circulating miRNA panels facilitate early detection, metastasis prediction, and therapeutic response monitoring.	Integration into exosomal miRNA liquid biopsy panels RNA-based therapeutic targets
lncRNAs/circRNAs	PVT1, circMTUS1	Control cell cycle and apoptosis Oncogenic regulation	Overexpression correlates with aggressive disease	Explored for early detection, disease tracking, and therapy-response monitoring Experimental lncRNA silencing strategies
Histone Modifications	EZH2, H3K27me3	Chromatin remodelling Transcriptional repression	Overexpression in metastases Absent in benign lesions	EZH2 inhibitors under clinical evaluation
Angiogenesis/ECM Remodelling	VEGF-A, TGF-β, Tenascin-C, LOXL3/4, COL6A	Promote angiogenesis, extracellular matrix remodelling, and metastasis	Elevated in metastatic UM Detectable in plasma	Anti-angiogenic or ECM-targeted therapies Potential monitoring markers
Immune Modulation	IL-10, CCL2, S100, M2 macrophage signatures	Create an immunosuppressive tumour microenvironment	Correlates with *BAP1* loss and immune evasion	Patient selection for immune-based therapies or clinical trials Immune-response monitoring

ALKBH5, alkB homolog 5; APC, adenomatous polyposis coli; BAP1, BRCA1-associated protein 1; CCL2, C-C motif chemokine ligand 2; CDKN2A, cyclin-dependent kinase inhibitor 2A; circMTUS1, circular MTUS1 RNA; COL6A, collagen type VI alpha chain; ctDNA, circulating tumour DNA; ECM, extracellular matrix; EMT, epithelial–mesenchymal transition; EZH2, enhancer of zeste homolog 2; FTO, fat mass and obesity-associated protein; H3K27me3, trimethylation of histone H3 at lysine 27; HINT2, histidine triad nucleotide-binding protein 2; IL-10, interleukin 10; lncRNA, long non-coding RNA; LOXL3/4, lysyl oxidase-like 3/4; METTL3, methyltransferase-like 3; miRNA, microRNA; M2, type 2 macrophages; PVT1, plasmacytoma variant translocation 1; RASSF1A, Ras association domain family member 1; RNA, ribonucleic acid; SF3B1, splicing factor 3b subunit 1; TGF-β, transforming growth factor beta; UM, uveal melanoma; VEGF-A, vascular endothelial growth factor A; ↑: increased; ↓: decreased.

**Table 4:** Biomarker-guided therapeutic decision pathways in ocular melanoma: clinical implications and monitoring strategies.

Biomarker	Molecular/Functional Role	Clinical Implication	Recommended Systemic/Targeted Therapy	Monitoring Strategy (Liquid Biopsy/Imaging)
**Uveal Melanoma**				
HLA-A*02:01 + gp100	Determines eligibility for TCR-directed therapy	Predicts response to tebentafusp	Tebentafusp ± immunotherapy	Serial ctDNA to assess early pharmacodynamic response Confirmatory radiologic imaging
*BAP1* loss ± Class 2 GEP	Immune-evasive microenvironment High-metastatic phenotype	High metastatic risk phenotype Candidate for immune modulation or adjuvant trials	ICIs (anti-PD-1/CTLA-4) or clinical trials	ctDNA/exosomal miRNAs for MRD detection Frequent imaging every 3–4 months
GEP Class 2	High-metastatic phenotype Strongly associated with *BAP1* inactivation	Strong predictor of metastasis	Local therapy + ICIs, targeted experimental treatment, or clinical trials.	Intensified imaging every 3–4 months; Longitudinal liquid biopsy surveillance
*SF3B1/SRSF2* mutation	Intermediate-risk Splicing alterations	Late-onset metastasis Potential RNA-targeted therapy	Experimental spliceosome inhibitors (clinical trials)	ctDNA mutation tracking Annual imaging
*EIF1AX* mutation	Low-risk Indolent phenotype	Standard surveillance only	Local therapy ± observation	Annual imaging Optional ctDNA monitoring
Detectable ctDNA (tumour-informed or driver mutations)	Indicates MRD or early metastasis	Trigger for systemic therapy escalation	Therapy per molecular subtype	Serial ctDNA trend analysis (Baseline and on-treatment)
**Conjunctival Melanoma**				
*BRAF* V600E	Activates the MAPK pathway	Predicts response to MAPK inhibitors	*BRAF* + MEK inhibitors ± ICIs	Clinical assessment + imaging Investigational ctDNA for treatment response
*NRAS* mutation	MAPK activation independent of *BRAF* Primary resistance to BRAF inhibitors	Poor prognosis; MEK-sensitive	MEK inhibitors ICIs (first-line)	Imaging ctDNA (NRAS) and exosomal miRNA monitoring LDH in blood
*NF1* loss	High TMB; enhanced immunogenicity	Predicts ICI responsiveness	ICIs (anti-PD-1/CTLA-4 or combination)	Imaging ctDNA + protein/cytokine panel exosomal miRNA monitoring
*C-KIT* mutation/amplification	Activates receptor tyrosine kinase signalling	Targetable in a subset of patients	Tyrosine kinase inhibitors (imatinib, dasatinib)	On-therapy imaging ctDNA/exosomal miRNA for recurrence or resistance monitoring
TERT promoter mutation	UV-related tumour aggressiveness	Prognostic marker potential therapeutic target	Experimental telomerase inhibitors	Combined ctDNA + methylated DNA monitoring

BAP1, BRCA1-associated protein 1; BRAF, B-Raf proto-oncogene, serine/threonine kinase; C-KIT, KIT proto-oncogene, receptor tyrosine kinase; ctDNA, circulating tumour DNA; EIF1AX, eukaryotic translation initiation factor 1A, X-linked; GEP, gene expression profile; gp100, glycoprotein 100; HLA, human leukocyte antigen; MRD: minimal residual disease; ICIs, immune checkpoint inhibitors; LDH, lactate dehydrogenase; MAPK, mitogen-activated protein kinase; MEK, MAPK/ERK kinase; miRNA, microRNA; NF1, neurofibromin 1; TMB: tumour mutational burden; NRAS, NRAS proto-oncogene, GTPase; PD-1, programmed cell death protein 1; SF3B1, splicing factor 3b subunit 1; SRSF2, serine/arginine-rich splicing factor 2; TCR, T-cell receptor; TERT, telomerase reverse transcriptase; CTLA-4, cytotoxic T-lymphocyte-associated protein 4; UV, ultraviolet.

## Data Availability

No new data were created or analysed in this study. Data sharing is not applicable to this article.
